# Impact of attention-deficit/hyperactivity disorder on the patient and family: results from a European survey

**DOI:** 10.1186/1753-2000-2-31

**Published:** 2008-10-28

**Authors:** David Coghill, Cesar Soutullo, Carlos d'Aubuisson, Ulrich Preuss, Trygve Lindback, Maria Silverberg, Jan Buitelaar

**Affiliations:** 1 Centre for Child Health, 19 Dudhope terrace, Dundee, Scotland, DD3 6HH, UK; 2 Child and Adolescent Psychiatry Unit, Clínica Universitaria, University of Navarra, Pio XII, 36. 31080-Pamplona, Spain; 3 Mühlenstrasse 61, 49324 Melle, Germany; 4 Universitätsklinik für Kinder-undJugendpsychiatrie Psychotherapie Bern, Effingerstrasse 12, CH-3011 Bern, Switzerland; 5 Ostadalsveien 58, 0753 Oslo, Norway; 6 överläkare, tf enhetschef, BUP Signal, Observatoriegatan 18, 113 29 Stockholm, Sweden; 7 UMC St. Radboud (966), Department of Psychiatry, Nijmegen, the Netherlands

## Abstract

**Background:**

Children with attention-deficit/hyperactivity disorder (ADHD) often experience problems with education, interaction with others and emotional disturbances. Families of ADHD children also suffer a significant burden, in terms of strain on relationships and reduced work productivity. This parent survey assessed daily life for children with ADHD and their families.

**Method:**

This pan-European survey involved the completion of an on-line questionnaire by parents of children (6–18 years) with ADHD (ADHD sample) and without ADHD (normative population sample). Parents were questioned about the impact of their child's ADHD on everyday activities, general behaviour and family relationships.

**Results:**

The ADHD sample comprised 910 parents and the normative population sample 995 parents. 62% of ADHD children were not currently receiving medication; 15% were receiving 6–8 hour stimulant medication and 23% 12-hour stimulant medication. Compared with the normative population sample, parents reported that ADHD children consistently displayed more demanding, noisy, disruptive, disorganised and impulsive behaviour. Significantly more parents reported that ADHD children experienced challenges throughout the day, from morning until bedtime, compared with the normative population sample. Parents reported that children with ADHD receiving 12-hour stimulant medication experienced fewer challenges during early afternoon and late afternoon/early evening than children receiving 6–8 hour stimulant medication; by late evening and bedtime however, this difference was not apparent. ADHD was reported to impact most significantly on activities such as homework, family routines and playing with other children. All relationships between ADHD children and others were also negatively affected, especially those between parent and child (72% of respondents). Parents reported that more children with ADHD experienced a personal injury in the preceding 12 months, including those requiring the attention of healthcare professionals. Although 68% of parents were satisfied with their child's current treatment, 35–40% stated that their child's ADHD symptoms needed to be more effectively treated during the afternoon and evening.

**Conclusion:**

This parent survey highlights the breadth of problems experienced by ADHD children and the impact throughout the day on both activities and relationships. Therefore, there is a need for treatment approaches that take into account the 24-hour impact of the disorder and include all-day coverage with effective medication.

## Background

Attention-deficit/hyperactivity disorder (ADHD), which is estimated to affect 4–12% of school-aged children, is one of the most common neurobehavioural disorders of childhood [1]. Although little doubt remains that ADHD affects both genders, the literature on ADHD in females remains limited [2]. ADHD is characterised by developmentally inappropriate levels of inattention, hyperactivity and impulsivity, which often gives rise to serious impairments in academic performance and social adaptive and behavioural functioning, both inside and outside the home [3,4]. Although ADHD symptoms have been shown to change with age (hyperactive and impulsive behaviour decreases, while inattention increasingly becomes predominant) [5], studies following children with ADHD into adolescence and early adulthood indicate that ADHD frequently persists and is associated with significant psychopathology, school and occupational failure, family and peer difficulties, emotional problems and low self-esteem [6-10].

ADHD is associated with an increased risk for accidents among children [11,12]. Compared to children without ADHD, children with ADHD were more likely to be injured as pedestrians (27.6% vs 18.3%, respectively) or bicyclists (17.1% vs 13.8%; respectively) and to have self-inflicted injuries (1.3% vs 0.1%; respectively) [11]. They were also more likely to have sustained injuries to multiple body regions (57.1% vs 43%; respectively), to have sustained head injuries (53% vs 41%; respectively) and to have been severely injured (13.5% vs 5.4%; respectively) [11]. During the past decade, epidemiological studies have also documented high rates of learning disorders and cormorbid psychiatric difficulties amongst children with ADHD, most commonly, oppositional defiant disorder and conduct and mood and anxiety disorders [13-15].

As they reach adolescence, children with ADHD are also at an increased risk for cigarette smoking and substance abuse [16-18]. Furthermore, a comparison between an ADHD sample of 239 consecutively referred adults with a clinical diagnosis of childhood-onset and persistent ADHD, and 268 non-ADHD adults, reported that subjects with ADHD were significantly more likely to make the transition from an alcohol-use disorder to a drug-use disorder (hazard ratio = 3.8) and were significantly more likely to continue to abuse substances following a period of dependence (hazard ratio = 4.9) [16].

Whilst debilitating for the child, ADHD has also been shown to adversely impact on parents' quality of life, placing a substantial burden on the family as a whole. Indeed, families of children with ADHD have been consistently shown to experience more difficulties than families of nondisabled controls [9,19]. These include disturbed interpersonal relationships, particularly less perceived family cohesiveness and greater conflict, depression in parents and higher incidences of divorce and separation [19]. In addition, childhood ADHD has been shown to adversely affect the child's parents' work status and work productivity. In a telephone survey of 154 caregivers of children diagnosed with ADHD, 63% of caregivers reported some change in their work status as a result of their child's ADHD. Of these, 15% changed their type of job, 46% reduced the number of hours worked per week and 11% stopped work completely [20]. In addition, during the 4 weeks prior to the survey, caregivers reported having lost an average of 0.8 days from work and being 25% less productive, for an average of 2.4 days, due to their child's ADHD [20].

Although the financial burden of ADHD has not been fully evaluated, it has been demonstrated that individuals with ADHD exhibit increased use of mental health, social and special education services [21,22]. Results from a population-based cohort study that compared medical care use and costs amongst 4880 children and adolescents with and without ADHD over a 9-year period, reported that the proportion requiring hospital inpatient, hospital outpatient or emergency department admission was higher for those with ADHD versus those without ADHD (26% vs 18% [p < 0.001], 41% vs 33% [p = 0.006] and 81% vs 74% [p = 0.005], respectively). In addition, median costs for all episodes of care during the 9 years of follow-up for persons with ADHD were more than double those of persons without ADHD ($4306 vs $1944, respectively; p < 0.001) [23].

The optimal management of ADHD aims to minimise not only the core symptoms, but also the associated impairments. Current practice suggests that children with ADHD benefit from medications such as stimulants (methylphenidate [MPH] and amfetamines) or the non-stimulant atomoxetine (Strattera^®^) [24,25], and that effective treatment requires a comprehensive multimodal approach that includes behaviour modification for many children [26]. MPH is the best-studied stimulant medication for ADHD, with results from a number of studies demonstrating that it significantly improves behavioural and attention-related symptoms of ADHD and academic and social functioning [27-32], as well as reducing sequelae such as the development of psychiatric disorders [32] and substance abuse [33]. The selective norepinephrine reuptake inhibitor, atomoxetine, has been shown to be effective in relapse prevention, with a suggestion that it may also have a positive effect on global functioning, specifically health-related quality of life, self-esteem and social and family functioning [34-36]. To date, much current research in ADHD has been focused on the objective management of symptoms, while the effect of the disorder on the everyday functioning and well-being of children with ADHD (e.g. the ability to undertake homework, participate in after-school activities and engage with friends and family) remains relatively unexplored [1]. To address this, a European parent survey was undertaken to examine the impact of ADHD on their children's everyday activities, general behaviour and family relationships, as assessed by parents. A secondary aim of the survey was to investigate the parental assessment of the effect of stimulant medication on the behaviours of their children with ADHD. This part of the survey was designed to have a particular focus on the early morning, afternoon and early evening period as this is the time when parents have the closest contact with their children.

## Methods

### Survey development and description

An on-line, parent-completed questionnaire was designed with input from both experts in the field of child psychiatry and paediatrics and experienced ADHD advocates. The primary aim of the survey was to examine the experiences of parents with a child with ADHD and the degree to which their child's ADHD impacts on both the daily life of the individual child and the family as a whole.

The survey was also designed to explore the differences in behaviour between children with ADHD receiving stable medication (> 3 months), children with ADHD not on medication and children without ADHD. As such, this questionnaire was completed by a sample of parents with children with ADHD and a general population sample of parents with children without ADHD (normative population). Parents of children with ADHD were questioned about the impact of their child's ADHD in three key areas: (i) everyday activities both 'in the home' (e.g. mealtimes and homework) and 'outside the home' (e.g. leisure and family activities); (ii) general behaviour (noisy or disruptive, aggressive or defiant, and impulsive or risk-taking behaviour); and (iii) family relationships (e.g. the relationships between the child with ADHD and their parents, siblings, peers and other adults). Similarly, parents in the normative population sample were questioned about their non-ADHD child's general behaviour and their behaviour in relation to everyday activities and family relationships.

All questions contained in the survey were multiple choice and answered using a 7-point scale. This survey also addressed the times of day at which the children were perceived by their parents to be affected by their ADHD (Table 1). On average, the time taken for parents of children with ADHD to complete the survey was 10 minutes; parents with non-ADHD children in the normative population sample took approximately 8 minutes to complete the survey.

**Table 1:** Times of day (including estimated start times) that the effects of ADHD were assessed

**Period of the day**	**Median start time**
Morning routine (waking up, getting ready for school)	07:00
Morning (school lessons)	08:00
Lunchtime	12:00
Early afternoon (lessons, homework and playtime)	14:00
Late afternoon/early evening	17:00
Late evening	20:00
Bedtime	21:00

Overall, parents from ten European countries (Belgium, France, Germany, The Netherlands, Norway, Poland, Spain, Sweden, Switzerland and the United Kingdom) were invited to participate in the survey. This survey was sponsored by Janssen Cilag and conducted by Harris Interactive, an experienced market research company. The survey was conducted in accordance with guidelines set by the European Pharmaceutical Market Research Associations and the Market Research Society.

### Sampling strategy

Parents of children with ADHD were drawn from an independently sourced sample via third party sample providers, who had identified households within their panel (which comprised a total of 1.2 million households in Europe) where one or more of the children in the household had ADHD (approximately 114,000 children across Europe). To supplement this sample group, additional respondents within the sample providers' panel were also invited to participate in a screening questionnaire. Parents of children with ADHD were surveyed between 1^st ^March to 11^th ^April, 2007.

A sample of parents with children without ADHD were drawn from the Harris pan European panel, which comprised of approximately 3 million households across Europe, and was representative of the general population within Europe in terms of age, gender and socio-economic status. Parents of children without ADHD were surveyed between 4^th ^June to 21^st ^June, 2007.

Both the ADHD and normative population surveys were conducted on-line and potential respondents were screened on a number of selection criteria. For the ADHD survey, parents were required to have a child (or children) aged 6–18 years, with a confirmed diagnosis of ADHD that had been made by a designated healthcare professional. Parents also had to live in the same household as the child with ADHD. Due to the fact that atomoxetine has a substantially different mechanism of action from stimulant medications, parents whose child with ADHD received atomoxetine were excluded from participating in the ADHD survey. In addition, given that one of the objectives of this survey was to investigate the impact of stimulant medication on afternoon behaviours, parents whose child with ADHD only received a once-daily dose of an immediate-release stimulant medication, were also excluded from the ADHD survey. In those instances where parents had more than one child with ADHD, parents answered questions with reference to their eldest child with ADHD. For the normative population survey, respondents also had to have a child (or children) aged 6–18 years and had to live with the child (or children). For parents with more than one child, the survey was completed with reference to the eldest child. In both surveys (ADHD and normative population surveys), data was collected and analysed for young (6–10 years) and older children (11–18 years). Gender was not considered as a specific issue during the design of the survey and as such gender was not controlled for in either the ADHD or the normative population survey samples.

**Figure 1:**
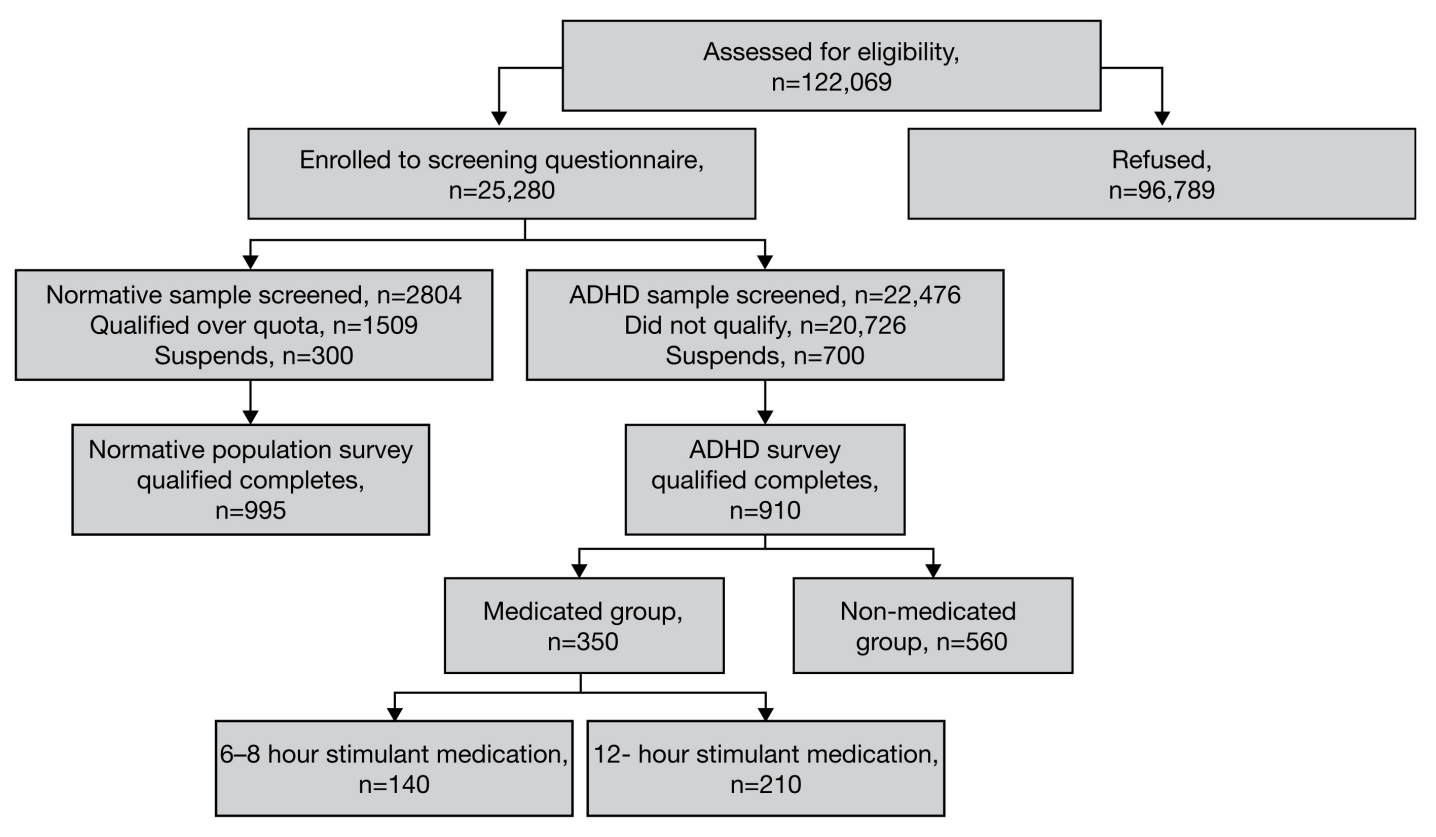
**Flow chart of survey design**. ADHD = attention-deficit/hyperactivity disorder.

Overall, invitations to participate in the survey were sent to 122 069 parents (104,018 parents with a child with ADHD and 18,051 parents without a child with ADHD), after which 25,280 parents were enrolled to the screening questionnaire. Following completion of the screening questionnaire, 910 parents were enrolled in the ADHD survey and 995 parents in the normative population survey (Figure 1)

### Statistical analysis

Data collected during the survey was analysed using parametric (t-test) or non-parametric (chi-square) tests as appropriate, carried out at the 5% significance level. Wincross (version 7.0) was used for this analysis. Data was analysed separately for the two groups (ADHD survey parent sample and normative population parent sample).

## Results

### Sample characteristics

Responses to the surveys were received from 1905 parents (ADHD parent sample, n = 910; normative population parent sample, n = 995) (Figure 1). The demographic and baseline characteristics of the responding parents and their children are provided in Table 2. The questionnaires were predominantly completed by mothers. As in most studies of ADHD, there was a strong male preponderance, with the majority of children described in the survey being boys (76% in both survey groups). Although it has been suggested that children with ADHD are over-treated, a large majority of children with ADHD in this survey (62%) were not currently receiving medication.

**Table 2:** Clinical characteristics of the ADHD and normative survey population

**Characteristics**	ADHD children (n = 910)*	**Normative children (n = 995)**^†^
**Responding parent**		
Female, n (%)	716 (79)	612 (61)
Male, n (%)	194 (21)	384 (39)
**Age of the majority of respondents, years**	38–47	40–44
**Marital status of respondents**		
Single/never married/widowed, n (%)	92 (10)	88 (9)
Married/cohabiting, n (%)	655 (72)	780 (78)
Divorced/separated, n (%)	160 (18)	128 (13)
**Number of children aged 6–18 years per household**		
1 child, n (%)	321 (35)	520 (52)
2 children, n (%)	375 (41)	333 (34)
>/= 3 children, n (%)	214 (24)	142 (14)
**Number of children with ADHD per household**		
1 child, n (%)	430 (73)	N/A
> 1 child, n (%)	159 (27)	N/A
**Gender of child**		
Male, n (%)	688 (76)	760 (76)
Female, n (%)	222 (24)	235 (24)
**Mean age of child, years**	11.4	12.0
**Average age of ADHD diagnosis, years**	6.4	N/A
**Medication status for child with ADHD**		
Receiving stimulant medication, n (%)	350 (38)	N/A
6–8 hour medication	140 (40)	N/A
12-hour medication	210 (60)	N/A
Not receiving medication, n (%)	560 (62)	N/A
**Length of time ADHD medication prescribed**		
3–6 months, n (%)	27 (8)	N/A
6–12 months, n (%)	52 (15)	
> 1 year, n (%)	271 (77)	

6–8 hour mediation consisted of long-acting medication taken once-daily or short short-acting medication taken twice-daily; 12-hour stimulant medication consisted of long-acting medication taken once-daily, short-acting medication taken three-times daily or a combination of long- and short-acting medication

*If > 1 ADHD child with ADHD in household, the survey was completed with reference to the eldest child

^†^If > 1 child without ADHD in household, survey was also completed with reference to the eldest child

ADHD = attention-deficit/hyperactivity disorder; N/A = not applicable

**Figure 2:**
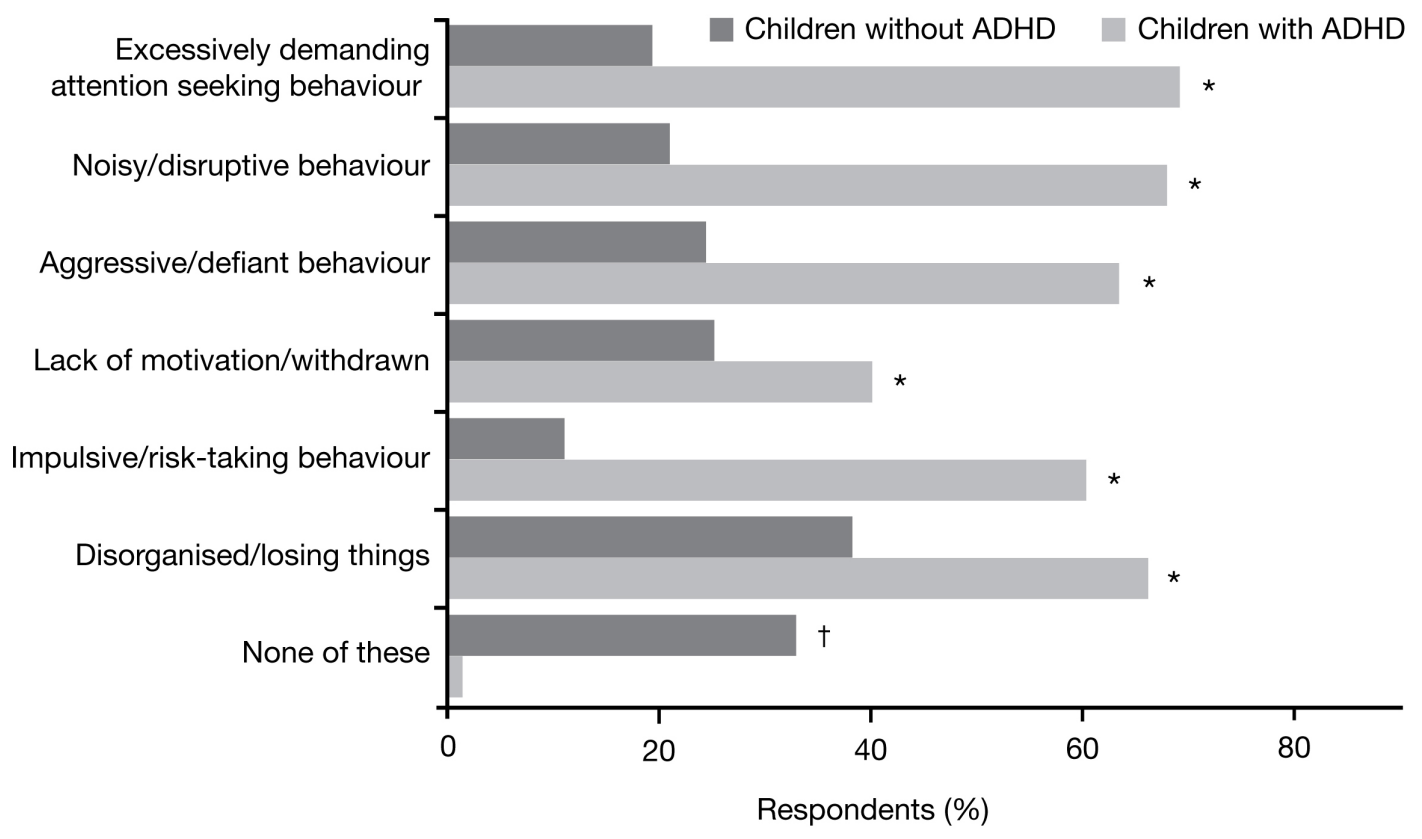
**Types of behaviour exhibited by children with ADHD compared to children without ADHD**. Baseline: all qualified respondents (ADHD survey, n = 910; normative population survey, n = 995). *p = 0.0001, non-medicated children with ADHD versus children without ADHD. ^†^p = 0.0001, children without ADHD versus non-medicated children with ADHD.

Figure 2 profiles the types of behaviour exhibited by the children as observed and described by their parents in the survey. Compared with the normative population sample, children in the ADHD sample consistently displayed more exaggerated behaviour as assessed by their parents.

With regards to the ADHD sample, an analysis of younger (6–10 years) versus older (11–18 years) children, revealed few differences on the impact of ADHD on everyday activities, general behaviour and family relationships as recorded by parents. Therefore, data is presented here for the entire ADHD age sample (6–18 years).

### Times of day that children with attention-deficit/hyperactivity disorder find challenging

Overall, parents reported that their children with ADHD find the whole day challenging. An analysis of parent's responses revealed that a reasonably high percentage of children with ADHD and children without ADHD experienced challenges with the morning routine (43% vs 41%, respectively; p = ns). However, over the course of the day, parents reported that children with ADHD consistently experienced greater challenges as observed during the morning (43% vs 12%, respectively; p < 0.05), at lunchtime (17% vs 3%, respectively; p < 0.05), during the early afternoon (50% vs 12%, respectively; p < 0.05), late afternoon/early evening (43% vs 12%, respectively; p < 0.05), late evening (33% vs 8%, respectively; p < 0.05) and at bedtime (38% vs 22%, respectively; p < 0.05).

When results were analysed for medicated versus non-medicated children with ADHD, a significantly higher percentage of children receiving stimulant medication experienced challenges with the morning routine compared with non-medicated children (55% vs 36%, respectively; p < 0.05). With the exception of the early afternoon period, where a greater percentage of non-medicated children experienced challenges compared with medicated children (53% vs 45%, respectively; p < 0.05), few other differences were observed by parents between the medicated and non-medicated ADHD groups during the course of the day.

**Figure 3:**
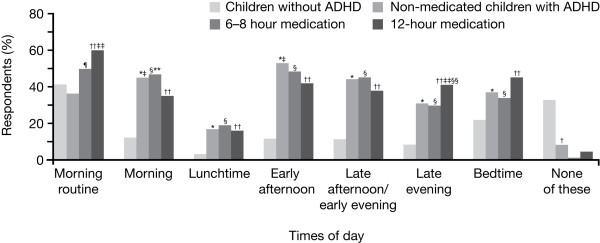
**Times of the day children with ADHD find challenging compared to children without ADHD**. Baseline: all qualified respondents (ADHD survey, n = 910; normative population survey, n = 995). ADHD = attention-deficit/hyperactivity disorder. *p < 0.05, non-medicated children with ADHD versus children without ADHD. ^†^p < 0.05, non-medicated children with ADHD versus 6–8 hours stimulant medication. ^‡^p < 0.05, non-medicated children with ADHD versus 12-hour stimulant medication. ^§^p < 0.05, 6–8 hour stimulant medication versus children without ADHD. ^¶^p < 0.05, 6–8 hour stimulant medication versus non-medicated children with ADHD. **p < 0.05, 6–8 hour stimulant medication versus 12-hour stimulant medication. ^††^p < 0.05, 12-hour stimulant medication versus children without ADHD. ^‡‡^p < 0.05, 12-hour stimulant medication versus non-medicated children with ADHD. ^§§^p < 0.05, 12-hour stimulant medication versus 6–8 hour stimulant medication.

For medicated children with ADHD, parents reported that those receiving 12-hour stimulant medication experienced greater challenges with the morning routine than those receiving 6–8 hour stimulant medication. However, as the day progressed, children receiving 12-hour stimulant medication experienced less challenges than children receiving 6–8 hour stimulant medication, although parents noted a trend for children receiving 12-hour stimulant medication to exhibit more challenging behaviour in the late evening and at bedtime (Figure 3).

### Everyday activities reported as challenging in children with attention-deficit/hyperactivity disorder

**Figure 4:**
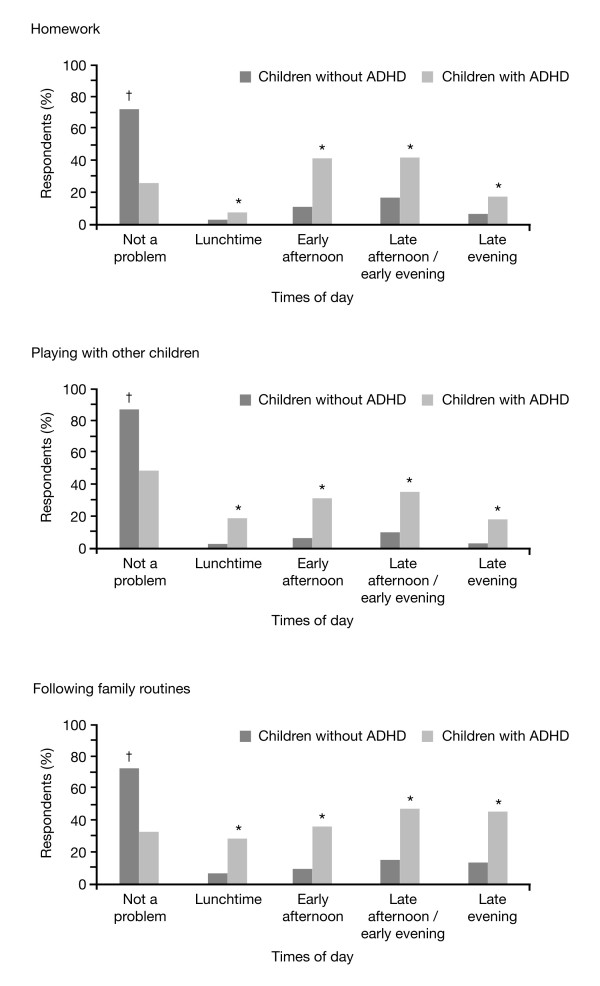
**Times of day activities are affected in children with ADHD compared to children without ADHD**. Baseline: all qualified respondents (ADHD survey, n = 910; normative population survey, n = 995). *p < 0.05, non-medicated children with ADHD versus children without ADHD. ^†^p < 0.05, children without ADHD versus non-medicated children with ADHD. ADHD = attention-deficit/hyperactivity disorder.

As part of this survey, parents were asked whether, on an average week day, their child's ADHD affected various everyday activities: meal-times, homework, playing alone, playing with other children, following family routines, individual leisure activities and group leisure activities. Overall, parents reported that ADHD is adjudged to impact negatively on all measured activities. In particular, compared with the normative population sample, a significantly higher percentage of children with ADHD were described as being considerably more challenged in the areas of homework (74% vs 28%, respectively; p < 0.05), following family routines (68% vs 28%, respectively; p < 0.05) and playing with other children (52% vs 13%, respectively; p < 0.05). When questioned at which times during the course of the day (lunchtime to late evening) these three activities were most affected in children with ADHD, parents reported that homework and playing with other children were most affected during the early afternoon and late afternoon/early evening, whilst following family routines was most affected during the late afternoon/early evening and late evening periods (Figure 4).

For children with ADHD, parental assessment on the impact of behaviour on everyday activities was found to be similar for both the medicated and non-medicated groups with a significant difference only being found for "playing with other children" (56% medicated vs. 49% non-medicated; p < 0.05). With regards to the times of day that homework and the following of family routines were most affected, a similar pattern emerged for both medicated and non-medicated children with ADHD, with parents reporting that both activities were most affected during the early afternoon and late afternoon/early evening periods. Playing with other children was reported by parents as being most affected during the early afternoon and late afternoon/early evening period. However, compared with non-medicated children with ADHD, a higher percentage of medicated children with ADHD experienced problems in the late afternoon/early evening periods as assessed by their parents (41% versus 31%, respectively; p < 0.05).

### Relationships affected in families with a child with attention-deficit/hyperactivity disorder

Overall, parents reported that ADHD impacted negatively on all relationships asked about: child-parent, parent-parent, child-sibling(s), child-other children and child-other adults. However, compared with the normative population sample, parents in the ADHD sample reported that the three relationships that were most affected were those between the child and parent (72% vs 43%, respectively; p < 0.05), the child and their sibling(s) (64% vs 29%, respectively; p < 0.05) and the child and other children (54% vs 12%, respectively; p < 0.05). When questioned at which times during the course of the day (lunchtime to bedtime) these three relationships were most affected, parents reported that they were affected over the whole time period assessed. Compared with the normative population sample, parents in the ADHD sample described the child-parent relationship as being most affected during the late afternoon/early evening (50% vs 24%, respectively; p < 0.05) and late evening periods (50% vs 21%, respectively; p < 0.05). Likewise, compared with the normative population sample, the child-sibling(s) relationship was also described by parents as being most affected in the late afternoon/early evening (52% vs 21%, respectively; p < 0.05) and late evening (41% vs 12%, respectively; p < 0.05) periods. Finally, compared with the normative population sample, the child-other children relationship was described by parents as being most affected in the early afternoon (41% vs 9%, respectively; p < 0.05) and late afternoon/early evening (38% vs 8%, respectively; p < 0.05) periods.

For children with ADHD, there was no effect of medication status on the relationships assessed, with parents reporting that the relationships between the child and parent, the child and their sibling(s) and the child and other children were similarly affected amongst medicated (73%, 67% and 57%, respectively) and non-medicated children with ADHD (71%, 62% and 52%, respectively). From lunchtime to early evening, there were no significant differences between medicated and non-medicated children with ADHD, in terms of the impact of their behaviour on the child-parent or child-sibling(s) relationship. However, compared with non-medicated children with ADHD, parents reported that a higher percentage of medicated children with ADHD experienced behavior that affected the relationship with their parents during the late evening (54% vs 47%, respectively; p < 0.05) and at bedtime (49% vs 40%, respectively; p < 0.05). Likewise, parents reported that the percentage of children with ADHD whose behaviour affected the relationship with their sibling(s) was significantly higher in the group receiving medication at bedtime (34% vs 27%, respectively; p < 0.05). Over the course of the day, no significant differences were reported by parents between medicated and non-medicated children with ADHD with regards to the impact of their behaviour on their relationships with other children.

### Different types of behaviours exhibited by children with attention-deficit/hyperactivity disorder

**Figure 5:**
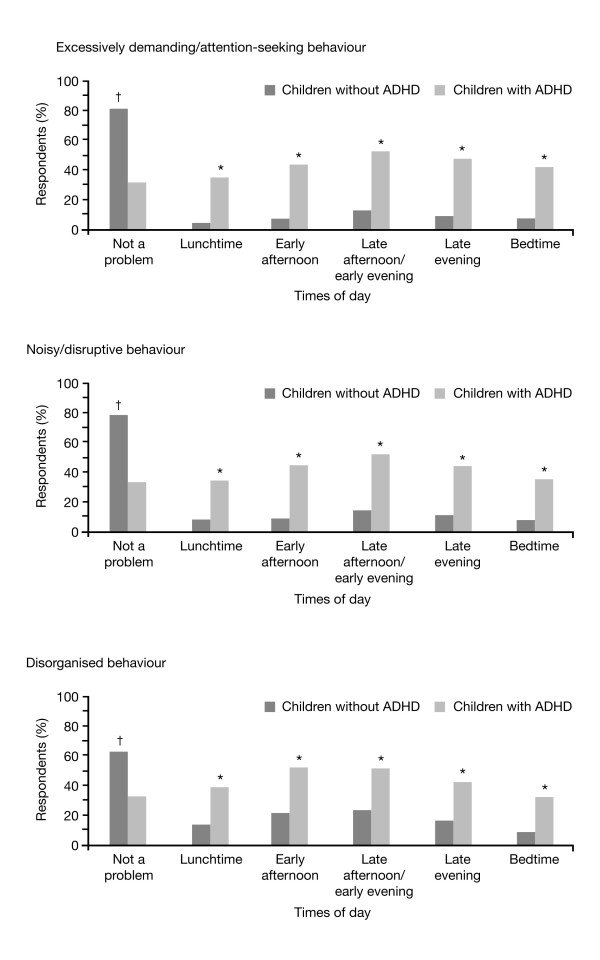
**Times of day certain behaviours are exhibited by ADHD children compared to children without ADHD**. Baseline: all qualified respondents (ADHD survey, n = 910; normative population survey, n = 995). *p < 0.05, non-medicated children with ADHD versus children without ADHD. ^†^p < 0.05, children without ADHD versus non-medicated children with ADHD. ADHD = attention-deficit/hyperactivity disorder.

When questioned about the types of behaviour displayed by their children with ADHD, parents reported a range of typical ADHD-related behaviours in their children (Figure 2). In particular, compared with the normative population sample, parents reported that children with ADHD displayed more noisy and disruptive behaviour (68% vs 21%, respectively; p = 0.0001), more disorganised behaviour (66% vs 38%, respectively; p = 0.0001) and more excessively demanding and attention seeking behaviour (69% vs 19%, respectively; p = 0.0001). When questioned at which times during the course of the day (lunchtime to late evening) such behaviours occurred in children with ADHD, a consistent pattern emerged, with parents reporting that such behaviours peaked during the late afternoon/early evening period, receding slightly during the late evening and at bedtime. A similar trend was also reported by parents in the normative population sample (Figure 5).

When results were analysed for medicated versus non-medicated children with ADHD, medication status did not significantly alter the proportion of children who exhibited typical ADHD-related behaviours. According to parental assessments, the percentage of children with ADHD reported to display noisy and disruptive behaviour, disorganised behaviour and excessively demanding and attention-seeking behaviour were comparable between the medicated (69%, 67% and 71%, respectively) and non-medicated groups (68%, 65% and 68%, respectively). With regards to the times of day that such behaviours occurred, parents reported that noisy or disruptive behaviour was displayed by similar proportions of medicated and non-medicated children with ADHD throughout the day, except at bedtime, when this was significantly more frequent in medicated children (39% vs 31%, respectively; p < 0.05). No significant differences in disorganised behaviour were reported by parents between medicated and non-medicated children with ADHD during the early afternoon, late afternoon and late evening periods; however, parents reported that disorganised behaviour was significantly more frequent in medicated children with ADHD at lunchtime (44% vs 36%, respectively; p < 0.05) and at bedtime (37% vs 28%, respectively; p < 0.05). Excessively demanding or attention-seeking behaviour was recorded by parents in a similar percentage of medicated and non-medicated children with ADHD over the course of the day.

### Number of personal injuries suffered by children with attention-deficit/hyperactivity disorder

Parents were also questioned about the number of personal injuries experienced by their children with ADHD. Compared with the normative population sample, parents reported that a significantly greater percentage of children in the ADHD sample (43% vs 28%, respectively; p < 0.05) experienced a personal injury in the last 12 months. In addition, an analysis of parent's responses suggest that children with ADHD experience a greater number of injuries that required the attention of a primary care physician or paramedic (1.5 vs 1.0, respectively; p < 0.05) and a visit to hospital (0.8 vs 0.6, respectively; p < 0.05).

**Figure 6:**
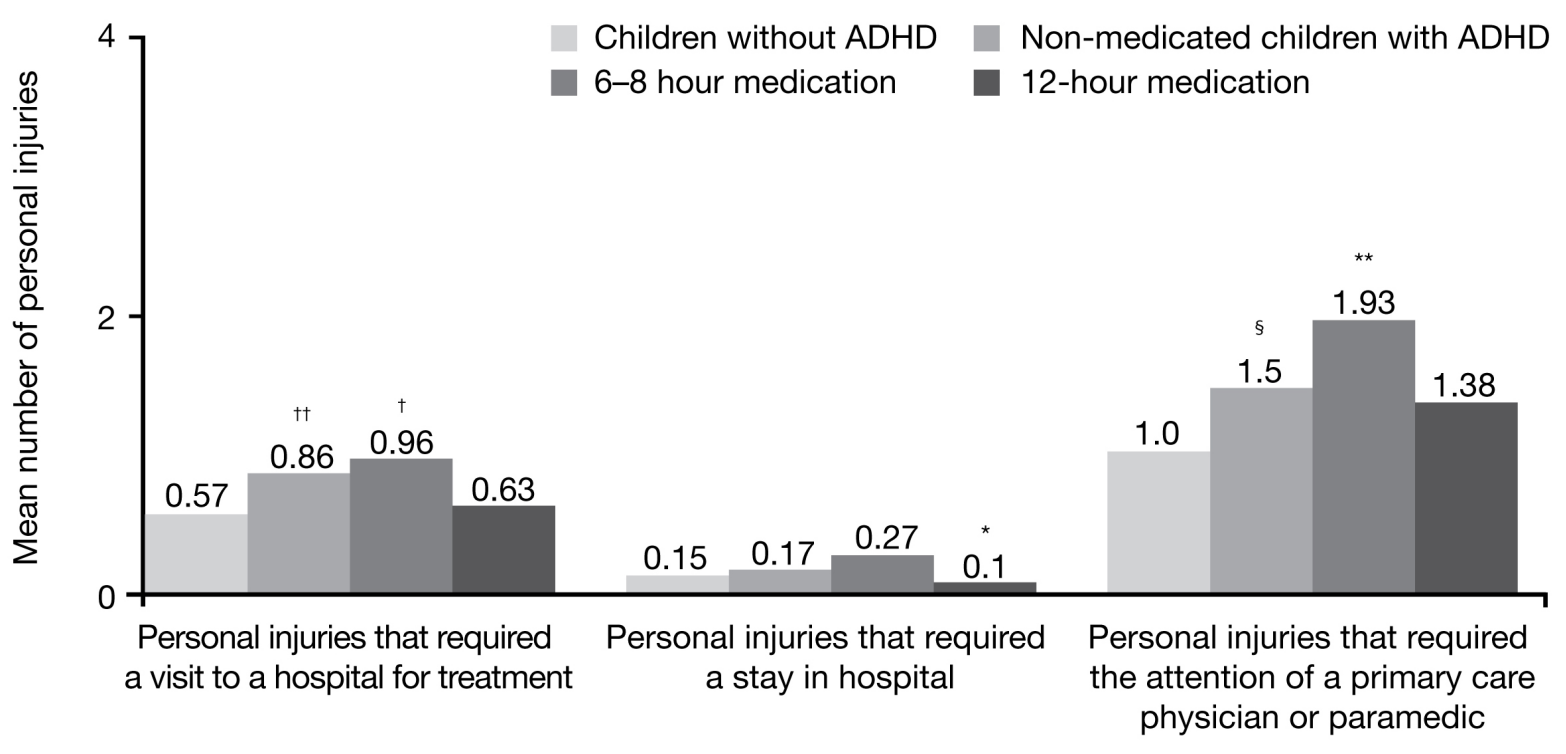
**Mean number of personal injuries in children with and without ADHD over the last 12 months**. Baseline: all qualified respondents (ADHD survey, n = 910; normative population survey, n = 995). *p < 0.05, 12-hour stimulant medication versus 6–8 hour stimulant medication.**p < 0.05, 6–8 hour stimulant medication versus children without ADHD. ^†^p < 0.05, 6–8 hour stimulant medication versus children without ADHD. ^†† ^p < 0.05, non-medicated children with ADHD versus children without ADHD. ^§^p < 0.05, non-medicated children with ADHD versus children without ADHD.

Medication status, as assessed by parents with an ADHD child, did not have a great impact on the number of injuries. Overall, there were no significant differences between medicated and non-medicated children with respect to percentage of medicated children with ADHD experiencing a personal injury in the last 12 months (39% vs 46%, respectively; p = ns), average number of total injuries (7.38 vs 6.87, respectively; p = ns), injuries that required the attention of a primary care physician or paramedic (1.60 vs 1.47, respectively; p = ns), injuries that required a visit to the hospital (0.77 vs 0.86, respectively; p = ns) or injuries that required a stay in hospital (0.17 vs. 0.17, respectively; p = ns). However when results for the injuries that required a stay in hospital were analysed separately for those receiving 6–8 and 12-hour stimulant medication, parents reported that children receiving 12-hour medication had significantly less injuries than those receiving 6–8 hour medication (0.1 vs. 0.27, respectively; p < .05)(Figure 6)

### Parents' attitudes towards medication for attention-deficit/hyperactivity disorder

Overall, the majority of children with ADHD (n = 562 [62%]) were not receiving medication at the time of this survey (Table 2). When questioned about the times of day (lunchtime to bedtime) when it was important for their child's ADHD symptoms to be medicated, 74% of parents felt that symptoms should be medicated in the early afternoon. In addition, 55% of parents also felt that it was important for symptoms to be medicated at lunchtime, while 54% also reported a need for medication during the late afternoon/early evening period.

Parents with an ADHD child treated with stimulant medication were also questioned about how satisfied they were with their child's current ADHD medication, using a 7-point scale ranging from 1 ('not at all satisfied') to 7 ('extremely satisfied').

Overall, parents were 'reasonably satisfied' with their child's medication, with 68% of parents recording a score of 5, 6 or 7 on this scale. When results were analysed for children receiving 6–8 hour stimulant medication and 12-hour stimulant medication, 71% of parents reported that they were reasonably satisfied with their child's 6–8 hour stimulant medication and 63% were 'reasonably satisfied' with their child's 12-hour stimulant medication, as recorded by a score of 5, 6 or 7 on this scale. Only 4% of parents (3% of parents whose ADHD child was receiving 6–8 hour stimulant medication and 5% of parents whose ADHD child was receiving 12-hour stimulant medication) recorded a score of 1, indicating that they were 'not at all satisfied' with the ADHD medication their child was receiving.

As part of this survey, parents with an ADHD child treated with stimulant medication were also asked whether there were any particular times of the day (lunchtime to late evening) when they felt that their child's symptoms needed to be better controlled. Overall, 47% of parents felt that their child's symptoms needed to be better medicated in the early afternoon. An additional 41% of parents felt that there was a need for improved medication in the late afternoon/early evening period, whilst 37% also reported a need for more effective medication during the late evening period.

## Discussion

Results from this large European parent survey demonstrate that parents report that ADHD has a significant impact on the child and their family, affecting school work, peer relationships and family relationships. Importantly, parental assessment in this survey clearly highlights that all times of the day are challenging for children with ADHD, with the afternoon/evening period at least as problematic as the school day. Of note, parents reported that typical behaviours associated with ADHD (excessively demanding/attention-seeking, noisy/disruptive and aggressive/defiant behaviour) consistently peaked during the late afternoon/early evening period. In line with this finding, a range of everyday activities (homework, playing with other children and following family routines) and a number of important relationships (child-parent, child-sibling and child-other children) were also reported by parents to be affected during the course of the afternoon and evening.

Of note, the majority of children with ADHD in this survey (62%) were not currently receiving medication. This may be due in part to a European tradition that medication treatments for ADHD should be reserved for those with more severe symptoms and impairments. This may also be related to a number of concerns that have arisen regarding the use of stimulant medication for children with ADHD, especially in younger children. These range from ethical objections to utilising medication to modify children's behaviour [37] to concerns about the lack of evidence for the long-term effectiveness of stimulant medication [38]. In addition, many ADHD children who were receiving pharmacotherapy in this survey continued to experience significant challenges over the course of a given day. Although medication status in children with ADHD was shown to have a positive impact on the number of personal injuries experienced in the last 12 months, parents reported that the proportion of children exhibiting typical ADHD-related behaviours and the impact of such behaviours on everyday activities were comparable between medicated and non-medicated children. Similarly, parents reported there was no apparent effect of medication on the relationships assessed, with relationships between the child and their parent, sibling(s) and other children equally affected amongst medicated and non-medicated children with ADHD. This may be due in part to individualised treatment plans for children with ADHD [39] and the successful implementation of a range of evidence-based psychosocial-intervention alternatives or adjuncts to pharmacological treatment such as educational interventions, intensive summer treatment programs, structure/routine and cognitive-behavioural therapy, social skills training and behavioural parent training [40-43]. Although results from a number of studies, including the Multi-modal Treatment Study of ADHD (MTA Study), have reported that medication management significantly improves ADHD symptoms [32], the applicability of these findings beyond the research settings into routine clinical practice remains less clear. Indeed, it has been suggested that in routine clinical care, where less intensive monitoring is available, many children with ADHD will not receive the maximum benefit from their medication [32]. This may account for the finding in this parent survey that many children with ADHD continued to experience challenges despite receiving prescribed medication for their ADHD. Another important consideration is the fact that these results are based on an observational study, in which allocation to treatment (i.e medication versus no medication) was not subject to experimental procedures such as randomisation. As such, the decision to prescribe medication will most likely have been made on the basis of clinician and parent judgment of the severity of ADHD symptoms and their associated impairment on functioning. Consequently, the most severely ill and impaired children with ADHD are likely to have been offered medication, a factor that may have resulted in the similarity of results between medicated and non-medicated children in this survey.

Although there is a misconception that ADHD is a condition that primarily affects children whilst at school (e.g. their school grades), which results in some physicians focusing solely on the impact of ADHD on school activities, results from this parent survey highlight the importance of treatment throughout the full active day. Indeed, when questioned directly, approximately half of parents reported the need for a medication that extends into the early afternoon and late afternoon/early evening periods. Moreover, the observation by parents that there was a trend for children with ADHD receiving 12-hour stimulant medication to experience more challenges during the morning routine, in the late evening and at bedtime may reflect that these children are those with the most severe ADHD, with pronounced disturbances late in the day, whose symptoms require medication coverage over the full course of the active day. However, it is important to note that individual treatment plans for children with ADHD may take into account not only the severity of symptoms and the ensuing daily challenges, but also the context (e.g. consequences and settings) of symptoms and the impact of these symptoms on daily functioning. Indeed, it has been suggested that the primary focus of assessment of ADHD should be on functional behavioural assessments of impairment such as identifying impaired domains of functioning, operationalizing target behaviours within these domains and implementing treatment measures such as Individualised Target Behaviour Evaluation. Such assessments will identify environmental contexts and socially valid target behaviours (not DSM-IV symptoms of ADHD) and facilitate treatment planning [39].

Interestingly, although specific parent enquiry demonstrated a significant degree of challenging behaviours, many parents who participated in this survey reported reasonable satisfaction with their child's current ADHD treatment. These results highlight that, by asking about specific activities at different times of the day, physicians may be able to elicit better information from parents that may guide individual medication decisions and optimize treatment across the day.

Given the importance of considering the treatment of ADHD symptoms outside of the school environment, there has recently been great interest in the use of longer acting stimulant preparations. Current international guidelines for the management of ADHD recommend the use of long-acting formulations to reduce the need for in-school dosage and the likelihood of diversion [44]. To date, a number of long-acting MPH formulations that allow once-daily dosing have been developed, including Equasym XL^®^, Ritalin LA^®^, Metadate CD^® ^and Concerta XL^®^. Results from a number of studies have demonstrated that these long-acting MPH formulations improve the behavioural and attention-related symptoms of ADHD over the course of the day [27-31]. In addition, the efficacy of these formulations has been shown to be comparable with that of immediate-release MPH, dosed three-times daily, in a number of double-blind studies [27,29,45]. Consequently, long-acting MPH preparations may have the potential to improve symptom control beyond the school day (i.e. extending into the late afternoon and early evening period), thereby ensuring the optimal personal development of children with ADHD. Although this survey found few parent-reported differences between those children on 6–8 hour medication and those on 12 hour medication this, as stated earlier, may reflect that the children on 12-hour medication were those whose symptoms were, when untreated, the most severe and long-lasting.

There are several limitations of this survey that must be noted. Due to the design of the survey, the core selection criterion for ADHD was based solely on the diagnosis made by a dedicated healthcare professional. Moreover, unlike clinical trials in which all attempts are made to standardise the research setting, surveys are conducted in the real world under circumstances that can not be fully controlled. In addition, because the data reported here are based on parental reports of the impact of their child's ADHD on everyday activities, general behaviour and family relationships, the accuracy of these reports may be subject to recall bias, subjective reporting by parents and other types of response errors. In particular, parents may have been unwilling to indicate whether their child engaged in behaviours contrary to the generally accepted norms of society, thereby reducing the reliability and validity of some of the results. Of note, parental assessment in this survey showed relatively small differences between medicated and non-mediated children with ADHD, in terms of the impact of their condition on everyday activities, general behaviour and family relationships, and the times of day reported by their parents as challenging. This may be due to the fact that this was an observational study and that the two ADHD groups (medicated and non-medicated ADHD children) were not matched for severity-of-illness. In addition, no information was available on the dosage of medication and medication compliance in the ADHD treatment group. Concerns regarding the use of stimulation medication for children with ADHD have been raised across Europe (e.g. ethical objections of prescribing medication to modify children's behaviour, lack of evidence for the long-term effectiveness of stimulant medication and concerns over the side effects of stimulant medication). This contributes to wide variations in prescribing practices across Europe. Consequently, comparisons between medicated and non-medicated children with ADHD observed in this naturalistic survey are somewhat difficult to interpret. A further limitation of this survey is that children with ADHD receiving the non-stimulant medication Strattera, or who only received one dose of an immediate-release stimulant medication, were excluded from the survey, and therefore the results presented may not generalize to these children. Overall, this was a large European survey with broad selection criteria, a factor that should be noted when interpreting the results. In the future, additional studies or surveys could employ different recruitment strategies and designs in order that they can provide clearer, accurate and precise results. In particular, future studies should consider the validity of the ADHD diagnosis and provide more stringent selection criteria. In addition, analyses designed to critically evaluate the impact, on the course and outcome of ADHD, of a range of variables including gender, severity-of-illness, dosage of medication and/or behavioral treatment and treatment compliance are required. Such analyses may help identify and predict which children with ADHD may respond more optimally to different treatments. However, despite these limitations, the data gathered during this parent survey provides clinicians with a wealth of information on the effect of ADHD and its medication on the everyday life of affected children and their families.

## Conclusion

Results from this parent survey demonstrates both the breadth of problems experienced by children with ADHD, as well as alerting physicians to a range of factors that should be considered in the management of children with ADHD. Importantly, by highlighting that children with ADHD experience challenges throughout the day, with the afternoon/evening period at least as problematic as the school day, this survey illustrates the importance of medication throughout the full active day. To assist both children with ADHD and their families, such medication should include a range of behavioural interventions as well as pharmacological treatments that can provide full-day coverage of symptoms. This will help children with ADHD achieve their full potential at home and at school, and with their families and friends. These results also reinforce the need for good quality medication management in order to derive maximum benefit from pharmacological treatments.

## Abbreviations

ADHD: attention-deficit/hyperactivity disorder; MPH: methylphenidate.

## Competing interests

DC has been an advisory board member for Cephalon, Eli Lilly, Janssen Cilag, Shire and UCB and has received research funding from Eli Lilly and Janssen Cilag.

CS is a consultant for Alicia Koplowitz Foundation, EINAQ, Eli Lilly, Janssen-Cilag, Juste, Otsuka/Bristol-Myers Squibb, Pfizer and Shire and has been a speaker for ADHI, Admirall-Prodesfarma, APNADAH, AstraZeneca, ASTTA, Bristol-Myers Squibb, Eli Lilly, Esteve, GlaxoSmithKline, Janssen, Novartis and Solvay. Cesar Soutullo has received research funding from Abbott, Alicia Koplowitz Foundation, Bristol-Myers Squibb, Eli Lilly, Navarra Department of Health, Novartis, Pfizer, Solvay, Shire, Spanish Department of Health, Stanley Medical Research Institute-NAMI and royalties from DOYMA, Editorial Médica Panamericana, EUNSA, Grupo Correo and Euro RSCG Life Medea.

CA has been a speaker for Janssen-Cilag and participated in a German Survey for Eli Lilly.

UP is a consultant for Swiss Medic and Canton Bern and has been a speaker for Eli Lilly, Pfizer, Novartis, Janssen-Cilag, Solvay Pharma, Vifor AG and Canton Bern. Ulrich Preuss has been an advisory board memeber for Eli Lilly, Janssen-Cilag, Lundbeck and Canton Bern and has research contracts with Eli Lilly, AstraZeneca and Canton Bern.

TL has been an advisory board member for Janssen Cilag.

MS has been an advisory board member for Janssen Cilag.

JB is a consultant, advisory board memeber and a speaker for Janssen Cilag BV, Eli Lilly, Bristol-Myer Squibb, UCB, Shire, Medice and Pfizer.

## Authors' contributions

All of the authors participated in a series of Advisory Board Meetings to assist with the design and development of the survey questionnaire. The authors worked together to analyse the results from the survey and interpret the findings. All authors participated in the development of the manuscript, including the writing and editing.

All authors proof read the manuscript and approved the manuscript prior to submission.
